# An Embodied Sonification Model for Sit-to-Stand Transfers

**DOI:** 10.3389/fpsyg.2022.806861

**Published:** 2022-02-17

**Authors:** Prithvi Kantan, Erika G. Spaich, Sofia Dahl

**Affiliations:** ^1^Department of Architecture, Design and Media Technology, Aalborg University, Copenhagen, Denmark; ^2^Neurorehabilitation Systems Group, Department of Health Science and Technology, Aalborg University, Aalborg, Denmark

**Keywords:** movement sonification, conceptual metaphor, music, rehabilitation, sit-to-stand, embodied cognition, auditory information display, kinematics

## Abstract

Interactive sonification of biomechanical quantities is gaining relevance as a motor learning aid in movement rehabilitation, as well as a monitoring tool. However, existing gaps in sonification research (issues related to meaning, aesthetics, and clinical effects) have prevented its widespread recognition and adoption in such applications. The incorporation of embodied principles and musical structures in sonification design has gradually become popular, particularly in applications related to human movement. In this study, we propose a general sonification model for the sit-to-stand (STS) transfer, an important activity of daily living. The model contains a fixed component independent of the use-case, which represents the rising motion of the body as an ascending melody using the physical model of a flute. In addition, a flexible component concurrently sonifies STS features of clinical interest in a particular rehabilitative/monitoring situation. Here, we chose to represent shank angular jerk and movement stoppages (freezes), through perceptually salient pitch modulations and bell sounds. We outline the details of our technical implementation of the model. We evaluated the model by means of a listening test experiment with 25 healthy participants, who were asked to identify six normal and simulated impaired STS patterns from sonified versions containing various combinations of the constituent mappings of the model. Overall, we found that the participants were able to classify the patterns accurately (86.67 ± 14.69% correct responses with the full model, 71.56% overall), confidently (64.95 ± 16.52% self-reported rating), and in a timely manner (response time: 4.28 ± 1.52 s). The amount of sonified kinematic information significantly impacted classification accuracy. The six STS patterns were also classified with significantly different accuracy depending on their kinematic characteristics. Learning effects were seen in the form of increased accuracy and confidence with repeated exposure to the sound sequences. We found no significant accuracy differences based on the participants' level of music training. Overall, we see our model as a concrete conceptual and technical starting point for STS sonification design catering to rehabilitative and clinical monitoring applications.

## 1. Introduction

Aided in no small part by advances in digital audio technology, sonification research has made considerable strides in the past two decades (Supper, 2015), and been found to have potential for application in several domains related to data exploration, process monitoring, motor training and assistive technology (Hermann et al., 2011; Minciacchi et al., 2020). Due to the versatility of modern sound synthesis platforms, it is now possible to build flexible and complex real-time auditory displays (Supper, 2015; Kantan et al., 2021). Of particular interest is the sonification of biological signals, which has been explored for rehabilitation (Guerra et al., 2020; Linnhoff et al., 2020), diagnostics (Ballora et al., 2004; Danna et al., 2013; Gionfrida and Roginska, 2017; Aldana Blanco et al., 2020) and monitoring (Aldana Blanco et al., 2020). In this study, we focused on sonifying the ubiquitous movement of rising from a chair—the *sit-to-stand (STS) transfer*.

*Movement sonification* has received an increasing amount of attention in recent years and shown significant therapeutic promise (Sigrist et al., 2012; Dyer et al., 2015; Guerra et al., 2020; Linnhoff et al., 2020). This has been bolstered by advances in computing power as well as inexpensive and portable motion-sensing hardware (Ma et al., 2016; Kos and Umek, 2018), not to mention some highly refined sensor fusion algorithms (Ribeiro, 2004; Madgwick et al., 2011). Movement sonification makes it possible to “hear” continuous kinematic information like the course of a trajectory or the velocity/position contour of a distal effector (e.g., limb), even during phases where the movement by itself otherwise generates no audible sound due to its low frequency nature (Effenberg, 2005; Vinken et al., 2013). There are direct cortical links between the auditory and motor regions of the brain, as well as a strong motor component involved in music listening through mechanisms of motor mimesis and resonance (Maes et al., 2014, 2016; Wallmark, 2014). Auditory displays are particularly suitable for perceptualizing biological time series information, since the omnidirectionality of sound reduces visual burden (Watson and Sanderson, 2001; Eldridge, 2006), the high temporal resolution of the auditory system facilitates temporal pattern discrimination, and humans are able to process multiple concurrent auditory streams (Eldridge, 2006; Hermann et al., 2011).

In motor learning applications, sonification can serve as means of providing task-relevant feedback in tandem with intrinsic feedback modalities such as vision and proprioception (Sigrist et al., 2012; Dyer et al., 2015; Effenberg et al., 2016; Dyer, 2017). Motor learning has been found to be more effective and stable when the brain is able to integrate multisensory feedback into a multimodal representation of the movement (as reviewed by Sigrist et al., 2012). For this to occur reliably, the temporal structure of the sonified feedback must correlate well with that of the intrinsic feedback (Parise et al., 2012). If this is achieved, the sonified feedback can theoretically *highlight* task-relevant information already present in intrinsic channels, facilitating sensorimotor associative learning below the level of conscious perception (Makino et al., 2016; Morone et al., 2021). Alternatively, sonification can be used as a form of sensory substitution, filling in for another task-critical sensory stream that is either damaged or missing, such as vestibular, visual or somatosensory loss in balance training (Dozza et al., 2007; Costantini et al., 2018).

In spite of all its potential, movement sonification has yet to find widespread adoption in rehabilitation contexts, partly because existing research fails to address important questions related to short- and long-term therapeutic effects, meaningful mapping design practices and aesthetic considerations (Guerra et al., 2020; Linnhoff et al., 2020). There are no guidelines for how sonified feedback should be designed (Dyer, 2017), and the development of generalized guidelines and sonic interactions is made difficult by the variability seen among patients and therapy paradigms (Lesaffre, 2018). Sonification has not been explored within STS training and monitoring to the same degree as in balance (Ma et al., 2016; Guerra et al., 2020) and gait training (Linnhoff et al., 2020), but we summarize the scanty existing literature. Nicolai et al. (2010) designed auditory biofeedback for supranuclear palsy patients, where their system measured forward trunk bend while sitting, and provided a sound cue to stand up when a threshold was exceeded. Partly owing to a small sample size, they were unable to detect significant improvements in STS assessments such as the Five Chair Rise (5CR) Test. In a small pilot study with healthy participants carrying out STS transfers, Wang et al. (2014) provided auditory feedback on STS, based on Kinect-based measurements. The feedback led to smoother head movements and larger minimum hip flexion angles, both of which were deemed positive by the authors. Music-based interventions have also been proposed and tested. Peng et al. (2011) applied music therapy (specifically Patterned Sensory Enhancement) to children with spastic diplegia, finding that the intervention led to greater knee and extensor powers, better *center of mass (CoM)* smoothness, and faster movement execution. Newbold et al. (2015) proposed a musical STS sonification scheme that mapped various STS parameters to melody, harmony, texture, and rhythm—however, they did not evaluate it in any way. On the whole, there is a lack of experimentally validated models and design guidelines for STS sonification catering to various patient groups and impairment types.

In this work, we focused on the general task of **communicating STS kinematic information through sound**, something that must occur in a stable, unambiguous manner in any rehabilitative or monitoring application. Specifically, we devised an adaptable sonification model for STS kinematics. The subsequent sections of this article provide details of its theoretical background, design, development, and final evaluation by means of a listening test experiment on 25 healthy participants.

## 2. Background

### 2.1. What to Sonify?

STS is one of the most common activities in daily life; its goal is defined as “moving the mass center of the body upward from a sitting to a standing position without losing balance” (Roebroeck et al., 1994). During STS, the body CoM initially moves forward in the horizontal direction, and this momentum is gradually transferred to the upward direction through coordinated rotations of the hip, knee, and ankle joints, which are dictated by co-contractions of the lower body and torso muscles (Roebroeck et al., 1994; Kerr et al., 1997). STS can be examined in terms of the trajectory of the CoM of the body, the involved joints (hip, knee, and ankle), body segments (head-arms-trunk, thigh, and shank), and several major lower limb muscles (Roebroeck et al., 1994). Modern inertial sensing technology has made it possible to extract an extensive amount of STS kinematic and kinetic data, making such sensors useful tools for STS performance assessment (Millor et al., 2014).

Raw inertial readings require extra signal analysis to derive meaningful, clinically relevant features (see Millor et al., 2014 for a detailed review). Several such features have been defined and captured using various configurations of accelerometers, gyroscopes, and magnetometers mounted at different body locations. An important one is the *transition duration* (time expended in performing STS), which can differentiate frail and healthy subjects (Millor et al., 2014). Several linear kinematic parameters have been found to differentiate pathological and non-pathological STS, such as vertical, mediolaterial and anteroposterior acceleration, anteroposterior jerk, and vertical linear velocity (Millor et al., 2014). Velocity and acceleration measures during STS have shown potential in geriatric screening as well (Shukla et al., 2020). There are also some clinically relevant angular kinematic parameters such as knee extensor velocity and trunk tilt (Millor et al., 2014).

We propose that clinically relevant parameters should be either sonified directly or easily deducible from the sonification of other parameters. Regardless of the approach or application, the function of the sonification is to serve as a communication channel for task-relevant information streams. Sound-based communication design for STS can thus take inspiration from communication design guidelines proposed for more conventional visual monitoring (O'Hara et al., 2004): (A) the displayed information should be synthesized to a high-level to reduce the required cognitive effort, and (B) the display should trigger an appropriate mental representation of the monitored situation *(STS characteristics)* using salient cues. The latter relates to the movement-sound mapping, which is discussed next.

### 2.2. How to Sonify?

Mapping choices are generally dictated by questions of veridicality (whether data relations can be heard correctly and confidently in the sounds), usefulness, usability and acceptance (Barrass and Kramer, 1999). Bakogiannis et al. (2021) suggest that *meaning* arises when the phenomenon (STS in this case) is represented using organized sound that enables the perception of information. They elaborate that a sonification model is well-designed when the representation is *structure-preserving* (preserves the main elements, connections and relations in the information) and *inference-preserving* (enables the listeners to draw conclusions about the phenomenon) (Bakogiannis et al., 2021). Despite the importance of these criteria, Supper (2015) argues that the topic of interpreting the meaning of sonified data has remained neglected in the sonification community, in favor of the development of technical solutions and tools (Supper, 2015). Consequently, no proven techniques exist for the design of veridical sonic representations of data in various contexts, including STS. Concerns related to aesthetics and usability are equally relevant. In rehabilitation settings, sonifications should be designed so as to minimize auditory fatigue (Dyer et al., 2015; Guerra et al., 2020). In monitoring settings, sonifications should be unobtrusive and compatible with the acoustic environment (Hildebrandt et al., 2014). In either case, it is important that the sound varies in tight connection with the underlying phenomenon, such that important and/or surprising state changes are conspicuous and clearly audible (Barrass and Kramer, 1999; Hildebrandt et al., 2014).

*Conceptual metaphor*-based approaches aim to address the problem of designing meaningful sonification schemes (Antle et al., 2009; Worrall, 2019; Roddy and Bridges, 2020). Theoretically, they do this by leveraging humans' existing embodied knowledge in familiar domains of thought in the task of understanding of an abstract domain (STS kinematics in this case). In other words, embodied associations built over a lifetime of experience with sound and music can be used to interpret information about movement kinematics, provided that the sonification mapping is designed accordingly (Roddy and Bridges, 2020). Music is the organization of sound into structures that most people are able to subconsciously decode without training (as in everyday music listening) (Vickers and Hogg, 2006), making it a good potential medium to provide sonified feedback (Newbold et al., 2015). Music and movement are closely connected (Maes et al., 2016), and musical characteristics such as melody, harmony, rhythm are often understood in terms of physical and spatial metaphors (Antle et al., 2009; Kelkar and Jensenius, 2018). The same goes for timbre, as listeners have been found to be able to recognize music genres within a fraction of a second (Mace et al., 2012) and derive meaning from timbal cues via motor mimetic processes (Wallmark, 2014).

Music is typically a rich and complex signal, and therefore not all of its structural properties have equally been explored for sonification purposes. Melodic sonification has been found to help in structuring and sequencing timed actions, along with recovering complex target patterns (Dyer et al., 2017). Interactive musical sonification can motivate, monitor and modify movement (particularly beat-synchronized rhythmic motion) (Maes et al., 2016), and allows comparable motor performance to non-musical sonification (Bergstrom et al., 2013). In some recent studies, metaphorical and musical sonification designs have been found to be advantageous, both in terms of information conveyance and aesthetic values (Antle et al., 2009; Danna et al., 2015; Roodaki et al., 2017; Aldana Blanco et al., 2020). In the STS sonification model proposed by Newbold et al. (2015), the central idea was that of using melodic phrases to represent STS movement phases within a pre-calibrated range of motion *(“exercise space”)*, with the pitch structure of the melody providing information of spatiotemporal progression within the phase. Melodic sonification has also been suggested for reaching exercises in chronic pain rehabilitation by Singh et al. (2014). Such a sonification design has the potential to convey STS parameters of clinical interest, such as the transition duration and relative durations of STS sub-phases. However, linear velocity, acceleration, and jerk information is less explicit in such a representation due to the spatial quantization that any melodic representation of position entails. Hence, it seems logical that other auditory dimensions such as timbre or dynamics must represent these parameters.

### 2.3. Framing the Present Work

Until now, embodied musical sonification schemes specific to STS have been proposed (Newbold et al., 2015) but, to the best of our knowledge, never been realized or evaluated in a rehabilitative or monitoring context. Given the widespread importance of STS, we believe that it would be of considerable value to devise a generic model to robustly communicate STS kinematic information. Due to the wide range of STS impairment types, the communication model must be adaptable to various use cases. In this study, we designed and developed a sonification model for STS based on embodied principles. Our goal was to create a model combining a melodic representation of the rising motion realized in a complex harmonic profile *(fixed component of model)* with salient sonic entities signifying movement impairments *(flexible component)*. To avoid ambiguities resulting from the complexities and culture-specific definitions and expectations related to music, we did not integrate any elements of rhythm and harmony into the present model. The model was evaluated by means of a forced-choice classification experiment, where healthy and cognitively unimpaired listeners were asked to identify simulated STS pattern types from their sonified sequences.

## 3. Materials and Methods

### 3.1. Sonification Platform and Design Guidelines

We used an upgraded version of the technological framework described by Kantan et al. (2021), with separate trunk-, thigh-, and shank-mounted M5Stack Grey ESP32 IMU devices wirelessly transmitting inertial data to a JUCE^1^ application for Windows. This software computes movement features in real-time and maps them to the synthesis parameters of physically-modeled musical instruments realized using FAUST DSP.^2^ These mappings are highly configurable in real-time in terms of topology, dimensionality, and mathematical transfer functions (Kantan et al., 2021). We modified the platform to enable the streaming, visualization and sonification of pre-recorded IMU data so as to generate multiple sonified versions of the same inertial data.

Note that the embedded IMU (MPU9250)^3^ and the ESP32 microcontroller are inexpensive and readily available. Hence the measurement setup is easy to reproduce. The JUCE framework is also free, and the sonification source code can be readily compiled for real-time use.

At the outset, we created a varied set of mapping configurations linking movement features (segment angles, joint angles, CoM horizontal/vertical displacement) to musical instrument model parameters (voice, guitar, piano simulations—pitch, dynamics, and consonance manipulations). These were targeted toward particular STS impairments that we compiled from literature (Kotake et al., 1993; Riley et al., 1997; Cheng et al., 1998; Scarborough et al., 2007) and through consulting with physiotherapists. We preliminarily evaluated the clinical potential of the mappings through expert interviews with five specialists (comprising physiotherapists, music therapists, and an experienced biofeedback researcher).^4^ By examining these disparate perspectives, we were able to formulate a broad design philosophy for STS sonification for feedback purposes, summarized as follows:

- “High-level” movement features (e.g., body CoM trajectories) are more directly coupled to the task (a.k.a. rising) and may be more suitable for sonification than “low-level” features (e.g., joint, segment angles) (similar suggestions made by O'Hara et al., 2004 for visual displays).
- The use of continuous sounds with no silent pauses will probably get fatiguing for patients in the medium/long term (as also explained by Roodaki et al., 2017).
- Physically-modeled musical instruments may be sufficiently pleasant-sounding to prevent auditory fatigue.
- Using an ascending melody to denote the rising motion may be a suitable representation (in line with the approach suggested by Newbold et al., 2015).
- The use of multiple musical instruments to concurrently represent separate movement features (see Video Demo 2—Mapping 2 in the interview materials) may be difficult for patients to understand.
- A potentially intuitive sonification design is:
  - No sound when the body is at rest.
  - Continuous movement features represented through continuous sound changes *(analogic mappings)*.
  - Discrete transient sounds to signify key *events* during the STS motion, e.g., when standing position is reached, instants of freezing *(symbolic mappings)*.

### 3.2. STS Movement Materials

STS impairments can be caused by age (Lord et al., 2002) as well as a range of neurological disorders and orthopedic problems/procedures (Millor et al., 2014). Some examples are stroke (Cheng et al., 1998; Boukadida et al., 2015), Parkinson's Disease (Mak et al., 2011), spastic diplegia (Peng et al., 2011), supranuclear palsy (Nicolai et al., 2010), and hip/knee arthroplasty (Wang et al., 2019, 2021), all of which require rehabilitation. Furthermore, older adults tend to use suboptimal chair rise strategies due to inefficient momentum transfer mechanisms (Scarborough et al., 2007). They may suffer from weakness or balance issues that manifest as “sitback failures” or “step failures” (Riley et al., 1997). Stroke patients exhibit decreased sensory ability and unilateral muscle weakness, leading to lateral deviation of the trunk, asymmetrical weight-bearing and extended rise times (Cheng et al., 1998; Boukadida et al., 2015). Post-arthroplasty patients show similar asymmetry (Wang et al., 2019, 2021). Parkinson's Disease patients suffer from bradykinesia and postural instability, leading to an increased fall risk (Mak et al., 2011) and children with spastic diplegia rise in a jerky and indirect manner (Peng et al., 2011).

Based on this knowledge and with the goal of sampling the wide set of existing STS impairments and assembling a set of distinct movement classes, we identified six STS patterns—see Table 1. Some of these were normal (P1) and others impaired, based on STS impairment literature (P3, P5, P6) (Riley et al., 1997; Scarborough et al., 2007; Peng et al., 2011; Millor et al., 2014; van Der Kruk et al., 2021) or our consultations with physiotherapists (P2, P4). However, the descriptions in Table 1 and experiment materials were derived from metronome-timed recordings of simulated movements by a healthy individual (the first author). Hence, these may not accurately mimic pathological STS patterns seen clinically among patients, but their purpose was to serve as movement material to test the informative potential of the sonification model. Video clips of the patterns are provided here.^5^

**Table 1:** Summarized description of the STS patterns P1-P6 in terms of their speed, stopping and jerkiness characteristics.

**Movement**	**(P1)**	**(P2)**	**(P3)**	**(P4)**	**(P5)**	**(P6)**
**attribute**	**Slow rise**	**Slow - fast**	**Failed attempts**	**Freezing**	**Jerky rise**	**Unstable ankles**
*Speed*	Uniform	Slow bend, fast rise	Multiple changes during bend	Multiple pauses during rise	Fast changes throughout	Uniform rise, fast changes post rise
*Stopping*	Once after rise	Once after rise	Once after rise	Multiple times	Once after rise	Once after rise
*Jerkiness*	None	None	Before rise	None	Entire	After rise

### 3.3. Movement Capture and Analysis

Based on Musić et al. (2008), we approximated the STS transfer using a three segment human body model in the sagittal plane. Although several studies have computed clinically relevant STS parameters using a single sensor (Millor et al., 2014), we chose to use three separate sensors (one per body segment) so as to obtain a more complete sagittal plane reconstruction of the STS movement (Musić et al., 2008), from which individual kinematic parameters could be estimated in a straightforward manner. In this model, the body position in the sagittal plane is uniquely defined in terms of the anteroposterior inclinations of the shank, thigh and HAT (head-arms-trunk) body segments, which in turn are used to compute the instantaneous CoM position of the body. The model uses anthropometric data (Drillis et al., 1964; De Leva, 1996) to estimate segment masses, lengths, and CoM positions. Several simplifying assumptions are made; the hip, knee, and ankle are considered frictionless pin joints, and the model is in contact with the ground only by the distal end of the shank. STS is assumed to be symmetrical in the frontal plane, although in reality this is often not the case in impaired individuals such as hemiparetic stroke patients (Cheng et al., 1998; Boukadida et al., 2015).

We briefly describe how our set of high-level movement features was derived from the sampled inertial data (3 axes each of accelerometer and gyroscope readings, 100 Hz) transmitted by the trunk, thigh and shank sensors. Upon reception, each axis underwent bias compensation by subtracting the axis-wise mean sensor reading over 10 s when the sensor was stationary, whilst accounting for the gravitational component. The result was processed with a 3-point median filter, and sensor fusion was then employed to compute the anteroposterior inclination of each segment. For this, we opted for the Madgwick Gradient Descent method (Madgwick et al., 2011) for its smaller error, superior computational efficiency and ability to operate at lower sampling rates than conventional Kalman filters. The error correction coefficient of the filter (β) was kept at its documented optimal value of 0.033 (Madgwick et al., 2011). Based on the segment angles and anthropometric data (Drillis et al., 1964; De Leva, 1996), the system was able to reconstruct the body trajectory in the sagittal plane.

**Figure 1:**
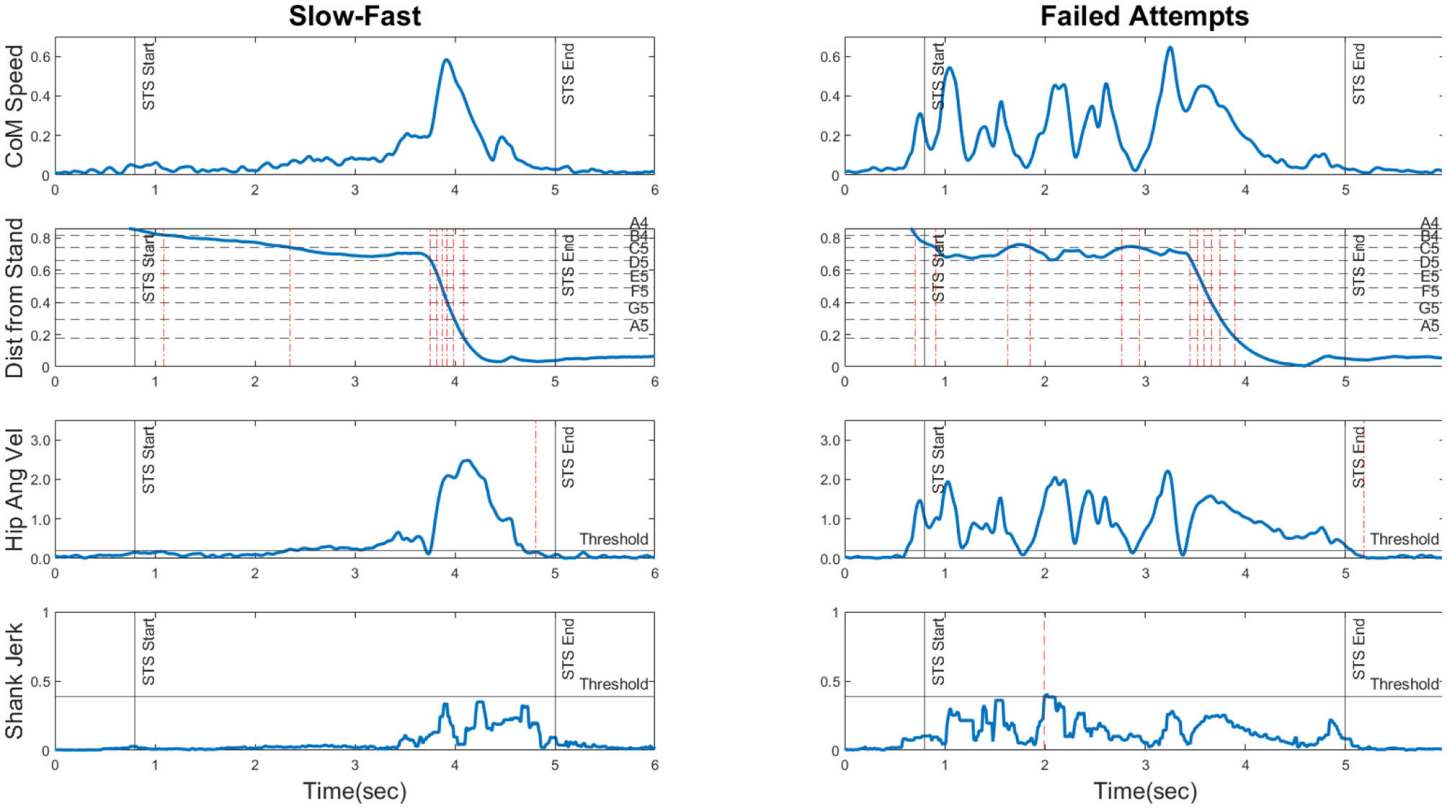
The four movement features (rows) plotted for two movement patterns (Slow-Fast and Failed Attempts) to illustrate their information content. The horizontal axis is time in seconds. *ROW 1: CoM Speed* shown in *bodylengths*/*s*. *ROW 2: Distance from Stand* shown as a fraction of the distance between the sit and stand coordinates. The horizontal dotted lines represent the distance thresholds corresponding to each note in the melodic pitch mapping (see Section 3.4). The vertical dashed-dotted lines represent instants of note transitions, providing a picture of the generated melodic contour. *ROW 3: Hip Angular Velocity* shown in *degrees*/*s*, and if it remains below the threshold for 130 ms consecutively, a freeze registered. Detected freezes are shown as vertical dotted lines. *ROW 4: Shank Jerk* shown as *degrees*/*s*^3^. The horizontal line “Threshold” represents the maximum permissible jerk threshold. The dashed lines represent positive threshold crossings. For corresponding plots of all six STS patterns, please refer to the Supplementary Material.

The next step was to periodically compute CoM position. First, we defined a relative coordinate system in the sagittal plane, placing the origin at the distal end of the shank. A unit distance (1) in these coordinates equalled the total length of the body with the hip and knee extended, and ankle in the neutral position (90° angle between shank and foot). The X and Y coordinates of each segment's CoM were computed from its inclination angle and the known position of its distal joint, starting from the ankle (0.0,0.0) and moving upwards:


$$\begin{array}{l} x_{seg}=x_{joint}+sin\left(\Theta_{seg}\right)\cdot L_{seg}\cdot D_{CoM}\\ y_{seg}=y_{joint}+cos\left(\Theta_{seg}\right)\cdot L_{seg}\cdot D_{CoM} \end{array}$$

where *x*_*seg*_ and *y*_*seg*_ represented the horizontal and vertical coordinates of the segment CoM, respectively, *x*/*y*_*joint*_ corresponded to the distal joint, Θ_*seg*_ was the segment inclination, *L*_*seg*_ was the length of the segment relative to total body height, and *D*_*CoM*_ was the fraction of the segment length between the distal joint and the segment CoM. The simplifying assumptions about the nature of the joints allowed the body CoM to be calculated as follows:


$$\begin{array}{l} x_{CoM}=x_{HAT}\cdot w_{HAT}+x_{thigh}\cdot w_{thigh}+x_{shank}\cdot w_{shank}\\ y_{CoM}=y_{HAT}\cdot w_{HAT}+y_{thigh}\cdot w_{thigh}+y_{shank}\cdot w_{shank} \end{array}$$

where *w*_*seg*_ represented the segment's weight as a fraction of the total body weight. These coefficients could be modified in the software to accommodate individual differences, but were configured based on normative data (Drillis et al., 1964; De Leva, 1996). CoM coordinate values corresponding to upright sitting and standing could be calibrated as well. The rectangular space in between these points was designated as the so-called STS “*exercise space”* (Newbold et al., 2015). The CoM- and body segment-related information was then used to extract the following features for sonification:

**CoM Speed:** Horizontal and vertical CoM velocity were obtained by single differentiation of *X*_*CoM*_ and *Y*_*CoM*_, and the instantaneous speed of the CoM was calculated as the Euclidean norm of these two vectors. This is shown for two STS patterns in the first row of Figure 1. For the *slow-fast* pattern, the contrast in CoM speed is clearly visible between the first and second halves of the rise duration. In the *failed attempts* pattern, the initial excursions represent multiple instances of trunk flexion and extension prior to rising.

**Distance from Stand:** This feature served as a continuous measure of STS progression, or “how far” the body was from a standing position, ranging from 1 (during steady sitting) to 0 (upright standing). To obtain this, the instantaneous Euclidean distance between the CoM position and the calibrated “stand” coordinates was first calculated. This was then normalized through division by the Euclidean distance between the calibrated “stand” and “sit” coordinates as follows:


$$\begin{array}{l} d=\frac{\sqrt{{\left(x_{stand}-x_{CoM}\right)}^{2}+{\left(y_{stand}-y_{CoM}\right)}^{2}}}{\sqrt{{\left(x_{stand}-x_{sit}\right)}^{2}+{\left(y_{stand}-y_{sit}\right)}^{2}}} \end{array}$$

This yielded the distance measure, depicted in the second row of Figure 1. For the *slow-fast* pattern, the gradient is initially shallow and suddenly steepens, corresponding to the slow bend and fast rise. In the *failed attempts* pattern, the repeated initial trunk flexions and extensions are seen as oscillations in the curve.

**Freezes During Rise:** This measure detected movement stoppages during STS by examining hip angular velocity, which was smoothed using a 2nd order Butterworth lowpass filter (fc = 7 Hz) and compared to an empirically set threshold (based on our recorded movement materials). If hip angular velocity remained below the threshold for a sustained period of 130 ms or longer, a *freeze* was registered. Note that this also occurred at movement completion in freeze-free STS. Freezes are shown as dashed vertical lines in the third row plots of Figure 1. Both movement patterns only exhibit one instance of movement stoppage, which occurs at the end of the rise.

**Shank Angular Jerk:** This was used to capture movement intermittencies, commonly seen in impaired movement patterns (Balasubramanian et al., 2011). Shank angular velocity was obtained from the gyroscope readings and double-differentiated along each axis to yield the angular jerk vectors, whose Euclidean norm served as the final feature value. This measure was chosen over acceleration-based linear jerk to avoid the influence of the gravitational component. A depiction is provided in the fourth row of Figure 1. The *slow-fast* pattern is relatively jerk-free until the fast rise, but the *failed attempts* pattern displays a greater degree of jerkiness during the initial portion of the rise.

Movement feature plots for all six STS patterns are shown in the Supplementary Material. We estimated the quantity of information necessary to reliably classify an STS pattern as a member of one of the six categories. In short, a combination of features appeared necessary, as no single feature seemed to clearly disambiguate all categories. *CoM Speed* trajectories of P1, P2, and P3 showed distinctive structural traits, but those of P4, P5, and P6 did not. *Distance from Stand* only exhibited distinctive contours for P2 (shallow initial gradient, sudden steepening) and P3 (pre-rise oscillations), whilst the others were less distinguishable. For our recorded data the *Freezes during Rise* feature seemed to allow the unique identification of P4, as it was the only pattern with multiple detected freezes. *Shank Angular Jerk* appeared to bring out differences among P3, P5, and P6 in terms of the temporal location and extent of jerky excursions. To summarize, we estimated for an STS pattern belonging to a random category, information of all four movement features would reliably allow the pattern to be identified.

### 3.4. Sonification Model

With that in mind, we developed a multidimensional model for the simultaneous sonification of all movement features. This was done using conceptual metaphors that represented the STS motion using a flute physical model *(fixed component of model)*, whilst saliently sonifying jerkiness and freezing when they occurred *(flexible component)*. We chose the flute for its continuous excitation signal, melodic capabilities, and mellow timbre. With most of its harmonic energy concentrated below 3 kHz, the sensitive 2–5 kHz range (Fletcher and Munson, 1933) could be occupied by the jerk and freezing feedback streams. An overview of the mapping principles follows, with technical specifics provided in Table 2. The first two mappings (S,P) are the fixed component of the model, whereas the last two (F,J) are the flexible component.

**Table 2:** A summary of mapping specifics.

**Short Form**	**Movement Feature**	**Mapped Perceptual Parameter**	**Param. Range**	**Mapping Polarity**	**Mapping Func. Order**	**Param. Smoothing Cutoff Freq**.
S	*CoM Speed*	Flute Blowing Pressure	0–1	+	0.45	6 Hz
P	*Distance from Stand*	Flute Melodic Pitch	A4–A5 note	-	0.75	No smoothing
F	*Freezes during Rise*	Bell Sound	Off–On	+	1	No smoothing
J	*Shank Jerk*	Flute Pitch Multiplier	1–10	+	0.45	19 Hz

*Mapping polarity refers to whether positive changes in the movement feature led to positive (+) or negative (−) changes in the perceptual parameter. Mapping function order refers to the shape of the transfer function linking movement feature values to perceptual parameter values. The latter may also have undergone smoothing via a 2nd order Butterworth Low Pass Filter, whose cutoff frequency (if applicable) is shown in the final column*.

**[S] CoM Speed** **→** **Flute Blowing Pressure:** The goal of this analogic mapping was to represent instantaneous energy changes in the movement as energy variations in the sound. Instantaneous velocity *v* correlates with kinetic energy of the body (*E* = 0.5·*m*·*v*^2^) (Fedak et al., 1982), and we therefore mapped CoM speed to the amplitude of the flute excitation signal. Sonically, this manifested as changes in volume of the tube resonances and the air blowing noise. The volume ranged from near-silence when the body is at rest (zero speed) to maximum intensity at the upper CoM speed bound.

**[P] Distance from Stand** **→** **Flute Melodic Pitch:** The STS progression (decreasing distance between CoM and stand coordinates) was discretely represented by the ascending tones of a major scale in a single octave. The distance feature received contributions from both the horizontal and the vertical CoM displacement components, meaning that forward trunk flexion also resulted in melodic pitch increases, although the vertical component was more dominant due to its relatively large contribution to the total distance traversed by the CoM.

**[F] Freezes During Rise** **→** **Triggered Bell Sound:** The goal was to symbolize freezes (discrete events) through discrete, salient auditory stimuli—specifically the sound of a church bell (physical model). Thus in freeze-free STS, the bell only sounded at movement completion, whereas it sounded multiple times during the course of freeze-ridden STS.

**[J] Shank Angular Jerk** **→** **High Pitch Modulations:** Jerky excursions during STS (typically manifesting as rapid, unpredictable speed changes) were sonified as salient high frequency pitch modulations. This can be seen as converting “glitchy” movements into “glitchy” sound. If shank jerk exceeded a threshold, a multiplicative factor was applied (proportional to threshold overshoot) to the fundamental frequency of the flute.

**Figure 2:**
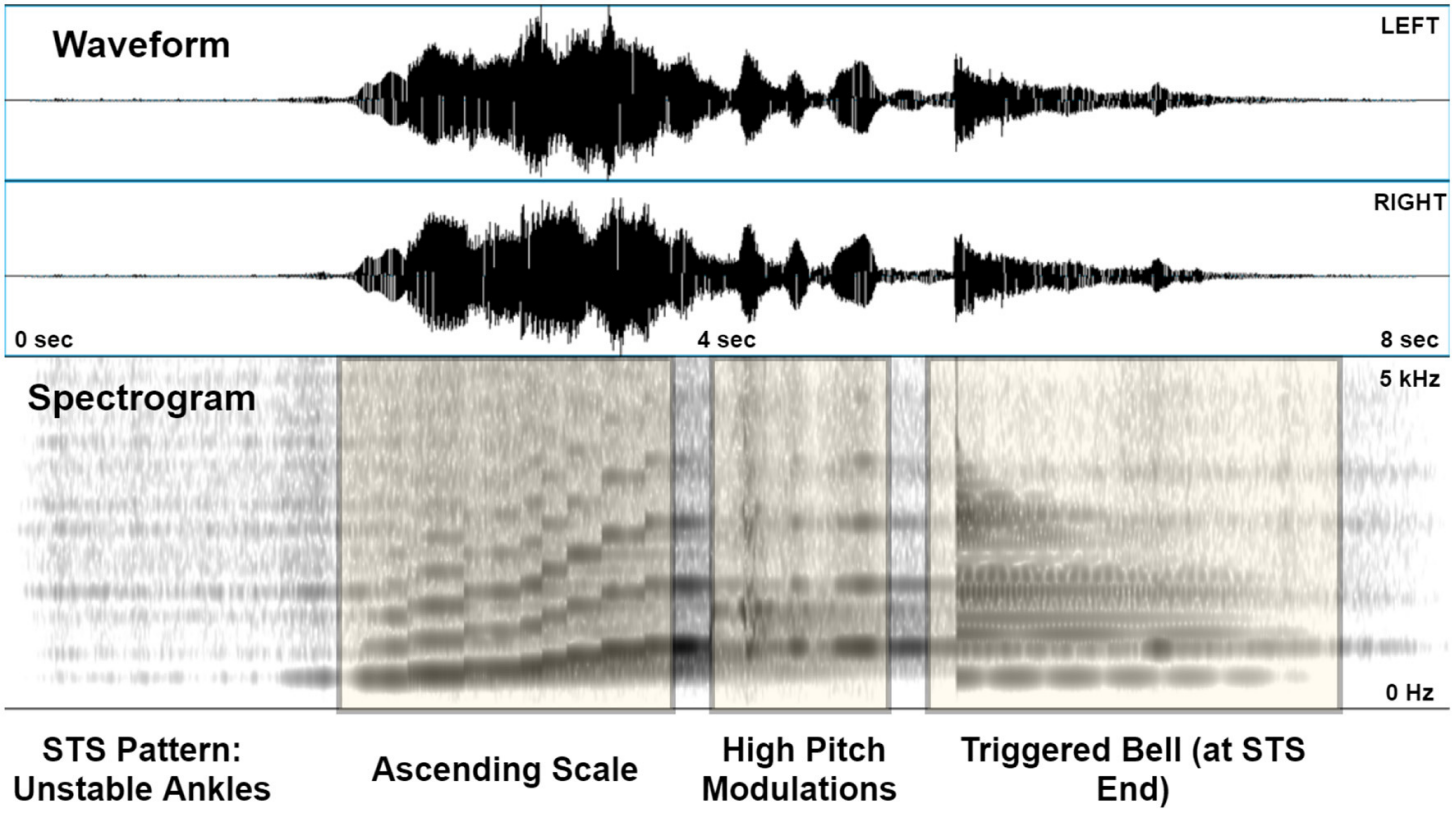
A sonified version of the “Unstable Ankles” pattern, shown as a stereo (2-channel) waveform and spectrogram (0–5 kHz, linear scaling) generated in PRAAT. The latter depicts the ascending scale, high pitch modulations post-rise, and the triggered bell on coming to rest after standing.

Figure 2 illustrates the audio signal generated by the *unstable ankles* movement pattern. Several key kinematic characteristics are apparent from the spectrogram. The ascending scale corresponding to the rise is clearly evident from the stepwise increases in fundamental and harmonic frequencies. The jerky excursions post rise are visible as pitch distortions, and the final stoppage of the motion is signified by the relatively broadband bell sound directly afterwards.

We estimated that information of all four movement features would theoretically enable STS pattern classification. As the communication medium in our case was sound, our estimation would only hold true if (A) the listener could understand how sound changes were linked to movement features, and (B) the sonic communication preserved the structure and integrity of the underlying information (Bakogiannis et al., 2021) in a way that was both perceptible and cognitively manageable. To test the model in these terms, we carried out an empirical evaluation in the form of a classification experiment inspired by Vinken et al. (2013).

## 4. Experiment

We applied a 6-alternative forced-choice paradigm where participants needed to identify STS movement patterns (see videos in Supplementary Material) by listening to sonifications generated using different combinations of the parameter mappings within the model. We aimed to assess how effectively the complete model could convey the movement information necessary to identify the movement patterns, in terms of classification accuracy, confidence and response time. Another goal was to assess the contribution of individual parameter mappings to the above outcomes, as well as the impact of music training.

### 4.1. Hypotheses

We formulated a list of concrete hypotheses based on findings from past literature:

- H1: Increasing the dimensionality of movement feature mappings contained will lead to an increase in classification accuracy and confidence, along with a decrease in response time. This applies particularly to the flexible component of the model (e.g., triggered bell and pitch modulations) (Barrass and Kramer, 1999; Bakogiannis et al., 2021).
- H2: STS patterns with distinct structural traits (e.g., failed attempts) that manifest across multiple movement features will be classified with greater accuracy, confidence, and speed than others (Vinken et al., 2013).
- H3: Repeated exposure to the sonified sequences over the course of the experiment will lead to learning effects that manifest as increased accuracy, increased confidence and decreased response time (Eldridge, 2006; Vinken et al., 2013).

### 4.2. Participants

A convenience sample of 25 participants (4 women) ranging from 22 to 49 y/o (30.28 ± 6.02 y/o) volunteered to participate in the study. They were primarily recruited from among the students and staff of Aalborg University, Copenhagen. None of them reported any auditory/cognitive impairment or any prior experiences with our experimental protocol, and they were all naïve about our hypotheses. All experimental procedures conformed to ethics code of the Declaration of Helsinki. The finalized experiment protocol was presented to the chair of Aalborg University's Research Ethics Committee, who approved the project on the basis that (1) informed consent was obtained prior to participation and (2) no personal, sensitive, or confidential information was collected from participants.

### 4.3. Materials and Setup

We generated sound sequences corresponding to multiple instances of all STS patterns, sonified using the parameter combinations shown in Table 3. One hundred and eight audio clips (3 instances of each movement type × 6 STS patterns × 6 parameter combinations) of equal duration (8 s) were generated. The sequences were edited and normalized in REAPER, and subsequently rendered as 48 kHz/24 bit WAV files. We have made these available in a Dropbox folder.^6^

**Table 3:** The six parameter combinations used to generate the sound sequences.

**Param. Combo**	**Flute Pressure**	**Flute Melodic Pitch**	**Bell Strike**	**Pitch Glitches**
	**CoM Speed (S)**	**Distance from Stand (P)**	**Rise Freezes (F)**	**Shank Jerk (J)**
S	✓			
SJ	✓			✓
SP	✓	✓		
SJF	✓		✓	✓
SPF	✓	✓	✓	
SJPF	✓	✓	✓	✓

*The abbreviations in the first column refer to the mappings present in that particular combination*.

The experiment was conducted with individual participants seated in a quiet room with no acoustic treatment. A Windows laptop running a custom-built experiment interface (built using JUCE) was used, and the sound sequences were played back over small USB-powered stereo speakers placed <1 m away, and at a comfortable volume. By using such a sound reproduction system and listening environment, the sound underwent comparable degradation to what it might be subjected to in real-world listening situations.

### 4.4. Procedure

Each participant was first given an overview of the purpose of the research and experiment. Next, the participant's Goldsmith General Musical Sophistication Index (GMSI) (Müllensiefen et al., 2014) was determined via an online questionnaire. The questionnaire consists of 29 items targeting self-reported music training, melodic and rhythmic ability^7^, yielding a score between 18 and 126 (Müllensiefen et al., 2014). Afterwards, the experiment was conducted over three separate phases: Familiarization, Training, and Testing.

**Familiarization Phase:** Participants watched two instructional videos (see Dropbox folder) that explained (A) the movement patterns, and (B) the individual and combined movement-sound mappings. The videos were formatted as text slides with information interleaved with audiovisual demos. Each movement pattern and mapping was shown once, but participants were instructed to carefully watch them as many times as necessary to familiarize themselves with the content. Once this was complete, the experimenter clarified any queries they may have had related to terminology and movement/mapping concepts. Info charts summarizing the STS patterns (identical to Table 1) and the movement-sound mappings were placed in front of participants for reference throughout the experiment.

**Training Phase:** This was carried out on the experiment interface (see Supplementary Material), where participants carried out the classification task on sound sequences from each of the six parameter combinations in Table 3, in reverse order starting from *SJPF*. The STS patterns were ordered based on a Latin squares design, and the participants had to attempt at least three classifications per parameter combination in order to proceed to the main experiment. The classification trials took on the same form throughout the experiment.

*Classification Trials:* Upon initiating a trial, a sound sequence was played back *once*, and there was no option to replay it. Upon its completion, the classification task was conducted as follows:

1. *STS Pattern Selection:* Participants were presented with six options (the STS patterns) and had to select the one that was first brought to mind by the preceding sound sequence. After clicking, they were unable to modify their selection.
2. *Confidence Rating:* They also had to report how *confident* they were in their choice by setting a slider value (0–100 integer value scale, 0 = not at all confident, 100 = fully confident).

Participants were allowed to refer to the info charts to help them finalize their choice, and there was no time limit for the task. After filling in their responses, they had to click a button to proceed.

In the training phase, the correct answer was displayed after each response, and participants were given the option of re-listening to the sound sequence while simultaneously watching a synchronized video clip of the correct pattern, so as to reinforce their understanding of the sound-movement relationship. Responses during this phase were not recorded. An illustration of the reinforcement learning interface is provided in the Supplementary Material.

**Testing Phase:** The six parameter combinations given in Table 3 were presented to participants over a series of six blocks, whose order was counterbalanced using a Latin squares design. At the start of each block, the interface informed participants of the new parameter combination. Within a block, classification trials were carried out with sound sequences corresponding to every STS pattern presented three times (order counterbalanced across blocks, consecutive sequences non-identical). This resulted in a total test length of *6 blocks* × *6 STS patterns* × *3 repetitions per pattern* = *108 classification trials*. The interface also recorded the time elapsed between sound sequence completion and STS pattern selection. On concluding a trial, 1-s white noise burst was played. This procedure was repeated until all 108 trials were complete, after which the experiment data was automatically logged. The experiment lasted a maximum of 2 h but was typically completed within an hour and a half.

### 4.5. Outcome Measures

1. *Classification Accuracy:* From the experiment logs, block- and pattern-wise responses were extracted and two measures of accuracy were computed in MATLAB 2018b (Mathworks):
  - **Percentage of Correct Responses:** We henceforth refer to this as Accuracy (%), computed as:
    $$\begin{array}{l} Accuracy\left(\%\right)=\frac{Correct\;Responses}{Total\;Responses}\cdot 100 \end{array}$$
  - **F-score:** This is the harmonic mean of precision and recall (commonly used in machine learning applications). It was calculated for each STS pattern as follows:
    $$\begin{array}{l} Precision=\frac{True\;Positives}{True\;Positives+False\;Positives}\\ \;Recall=\frac{True\;Positives}{True\;Positives+False\;Negatives}\\ \;FScore=\frac{2\cdot Precision\cdot Recall}{Precision+Recall} \end{array}$$
    When analyzing accuracy in terms of individual STS patterns, the F-score was the most suitable metric because high precision (fewer false positives) and high recall (fewer false negatives) are both clinically important, and the F-score gives them equal weight.
2. *Confidence:* This was a measure of uncertainty in the classification process, and was rated by participants during each trial (ranging from 0 to 100%).
3. *Response Time:* The elapsed time between the end of the sound sequence and the instant an STS pattern option was clicked, as recorded by the interface.

### 4.6. Statistical Analysis

All statistical analysis was done in SPSS 27.0 (IBM Corp). Summary measures for the data are presented as **mean** ± **standard deviation**. *Repeated measures (RM)* ANOVAs were used to test for effects of *Parameter Combination* and *STS Pattern* on classification accuracy, confidence and response time **(H1 and H2, respectively)**. Overall F-scores for each STS pattern were also computed within every *parameter combination*, along with confusion matrices. To investigate learning effects **(H3)**, the following analyses were carried out:

- *Across-Block Effects:* The *Block* factor represented the temporal progression of the experiment. RM ANOVAs were used to test for effects of *Block* on accuracy (%), confidence, and response time.
- *Within-Block Effects:* This refers to learning effects brought about by repeated exposure to the sound sequences (three repetitions of each STS pattern per block). An RM ANOVA was used to test the effect of sound sequence repetition *(Repetition Number)* on participant accuracy (%). The 36 sound sequences (6 STS patterns × 6 parameter combinations) were then analyzed in terms of elicited confidence and response time across repetitions. The sequences were first clustered using the k-means algorithm (k = 2) due to large variability in the data. *3 Repetition Numbers* × *2 Clusters* mixed-design RM ANOVAs were then used to test within-block learning effects and compare them across clusters.

In addition, some secondary analyses were conducted:

- *Confidence-Response Time Relationship:* This was investigated using a two-tailed correlation analysis between rated confidence and recorded response time over the 2,700 total responses (25 participants × 108 responses).
- *Effects of Music Training:* We first ran a two-tailed correlation analysis between participants' overall accuracy (%) and their GMSI scores. Next, an RM ANCOVA was carried out with *Parameter Combination* as the within-factor and *GMSI Score* as the covariate to measure the effect of music training on each outcome.
- *Differences between Correct and Incorrect Responses:* Confidence and response time behavior were compared between correct and incorrect classification responses. To minimize the impact of between-participant variability on these analyses,both outcomes were first normalized through division by the respective participant's grand mean (over the entire experiment) and expressed as a percentage. One-way ANOVAs were then carried out.

For all statistical tests, we used a significance criterion α = 0.05. If significant main effects were detected by the ANOVAs, Tukey *post-hoc* pairwise comparisons were carried out with Bonferroni correction applied. The JUCE code, data logs, analysis scripts, and SPSS test outputs are available on GitHub.^8^

## 5. Results

We found that participants were able to carry out the classification task with an overall mean accuracy (%) of 71.56%. This was well above chance level accuracy in a 6-alternative forced choice paradigm (16.67%). The overall rated confidence was 64.95 ± 16.52% and response time per trial was 4.28 ± 1.52 s.

### 5.1. Effect of Parameter Combination

**Figure 3:**
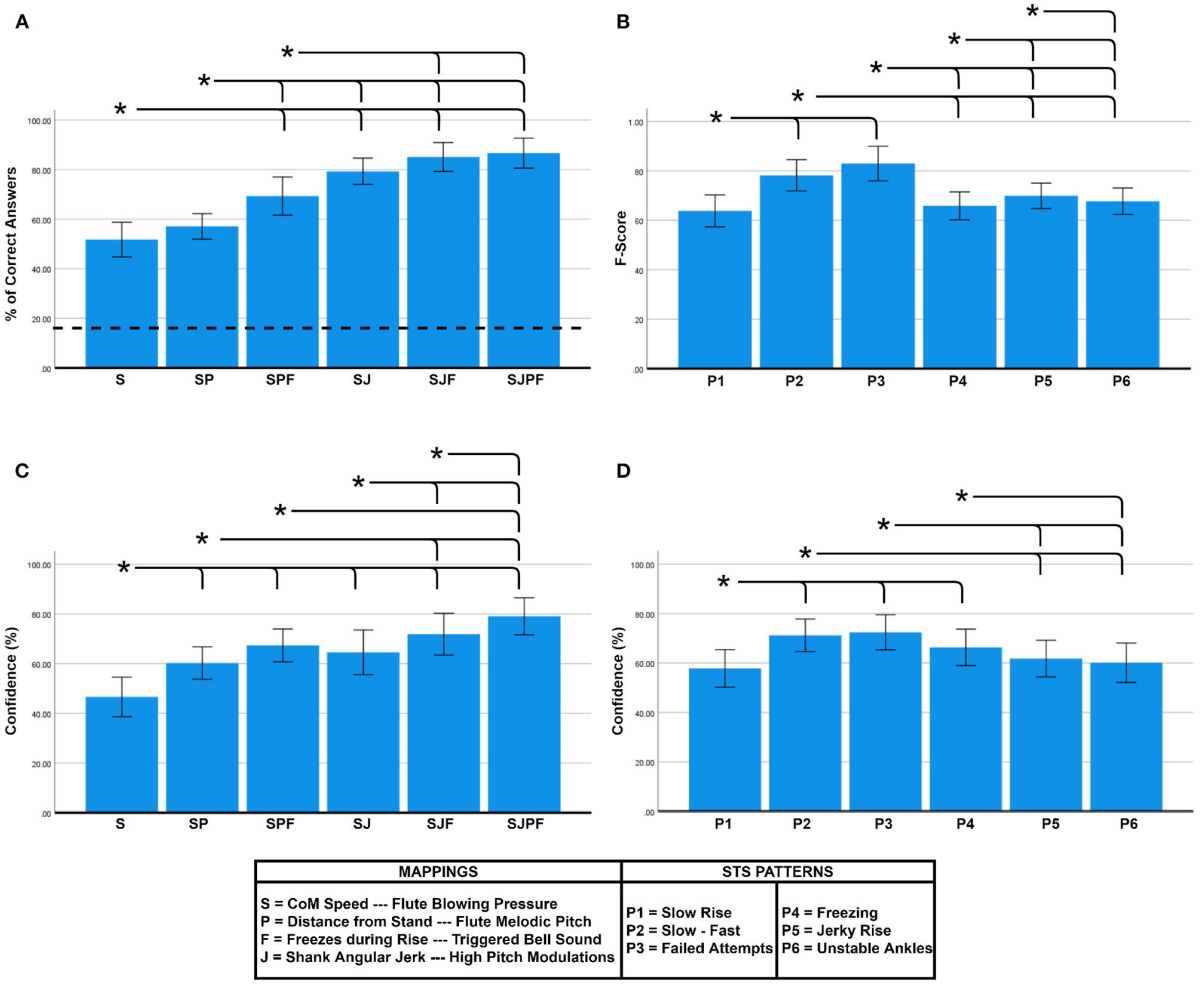
**(A)** Accuracy comparison between parameter combinations, **(B)** F-score comparison between STS patterns, **(C)** Confidence comparison between parameter combinations, and **(D)** Confidence comparison between STS patterns. Bar heights indicate mean values, and the error bars show 95% confidence intervals. Asterisks indicate significant pairwise differences (α = 0.05). Dotted line in **(A)** signifies chance level accuracy (16.67%).

Figure 3A shows the accuracy (%) within each parameter combination. The RM ANOVA showed a significant main effect of *Parameter Combination* on accuracy [*F*_(5, 120)_ = 40.586, *p* < 0.001, $\eta_{p}^{2}=0.628$]. Tukey *post-hoc* tests found multiple significant pairwise differences (shown as brackets in Figure 3A). As can be seen, accuracy was well over chance level in all cases, and showed a clear increasing trend with the addition of individual mappings. The highest accuracy (86.67 ± 14.69%) was found for the combination sonifying all four movement features—CoM Speed, Distance from Stand, Freezes, and Shank Jerk *(SJPF)*. At the opposite end, only sonifying CoM speed *(S)* resulted in the lowest accuracy (51.78 ± 16.95%).

In terms of rated confidence, Figure 3C shows an increasing trend with the addition of feature mappings. The RM ANOVA revealed a significant main effect of *Parameter Combination* on confidence [*F*_(5, 120)_ = 33.392, *p* < 0.001, $\eta_{p}^{2}=0.582$], with *post-hoc* comparisons revealing several pairwise differences (see Figure 3C). *SJPF* (79.08 ± 18.04%) elicited significantly higher confidence than all other combinations, and *S* (46.60 ± 19.27%) was significantly lower than all others, with the intermediate combinations all lying in between these extremes. There was also a main effect of *Parameter Combination* on response time [*F*_(5, 120)_ = 4.578, *p* = 0.001, $\eta_{p}^{2}=0.160$], although *post-hoc* comparisons did not reveal any significant pairwise differences. We discovered a significant negative correlation between participant-normalized confidence and response time values (ρ = −0.395, *p* < 0.01).

### 5.2. Effect of STS Pattern

As shown in Figure 3B, there was considerable variability in the measured F-scores between the STS patterns. The RM ANOVA revealed a significant main effect of *Pattern* on accuracy (F-score) [*F*_(5, 120)_ = 14.950, *p* < 0.001, $\eta_{p}^{2}=0.384$]. *Post-hoc* pairwise comparisons revealed multiple pairwise differences (see Figure 3B). *P2 (slow-fast)* (0.78 ± 0.15) and *P3 (failed attempts)* (0.83 ± 0.17) exhibited significantly higher F-scores than all other patterns. A significant main effect of *Pattern* on confidence was also found [*F*_(5, 120)_ = 16.708, *p* < 0.001, $\eta_{p}^{2}=0.410$]. *Post-hoc* comparisons revealed similar trends to the F-score plot (see Figure 3D).

**Figure 4:**
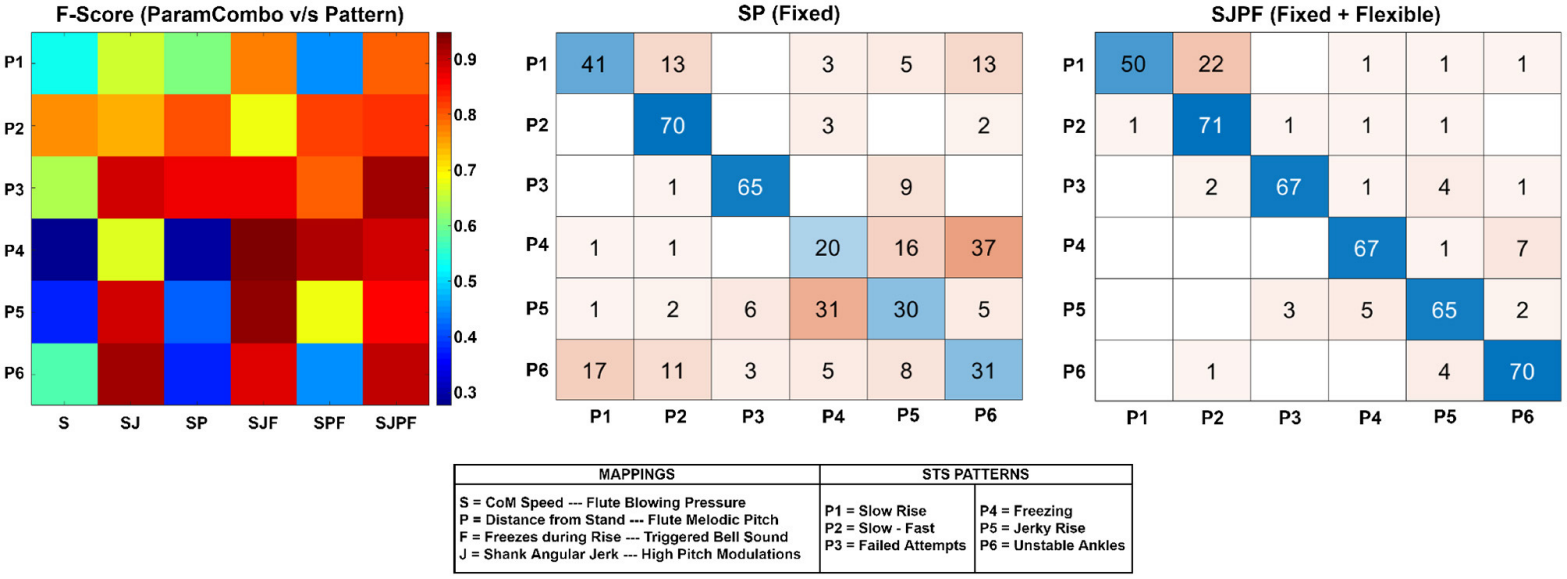
**Left:** Heatmap depicting F-score distribution across patterns P1-P6 (vertical direction) and parameter combinations. Higher values indicate superior performance. **Middle:** The confusion matrix for the *SP* parameter combination (fixed component of model). Rows represent true classes, and columns represent predicted classes. Thus, the diagonal elements represent correct classifications, and the rest are incorrect. **Right:** Confusion matrix for the *SJPF* parameter combination (fixed + flexible components of model).

The F-scores for each pattern and parameter combination are visualized as a heatmap in Figure 4 (left). In general, an increasing tendency (progressively redder hues) is visible from left to right as the mapping dimensionality increases, and the tendency is highly pattern-dependent. In the middle plot, the confusion matrix for the *SP* parameter combination *fixed component of sonification model* is shown. Patterns P4 *(freezing)*, P5 *(jerky rise)*, and P6 *(unstable ankles)* were the patterns most commonly misclassified and confused with one another. Figure 4 (right) shows the confusion matrix for *SJPF (complete model)* (rightmost column of heatmap), which had far fewer misclassifications overall. 22/60 total misclassifications were instances of P1 *(slow rise)* wrongly marked as P2 *(slow-fast)*, with sporadic incidences of other miscellaneous confusions. Confusion matrices for the remaining parameter combinations can be found in the Supplementary Material.

### 5.3. Learning Effects—Within- and Across-Block

The RM ANOVA revealed no significant effect of *Block* on accuracy [*F*_(5, 120)_ = 0.799, *p* = 0.553, $\eta_{p}^{2}=0.032$], confidence [*F*_(5, 120)_ = 0.401, *p* = 0.847, $\eta_{p}^{2}=0.016$], or response time [*F*_(5, 120)_ = 1.418, *p* = 0.222, $\eta_{p}^{2}=0.056$].

In terms of within-block effects, the RM ANOVA showed a significant main effect of *repetition number* [*F*_(2, 48)_ = 7.865, *p* = 0.001, $\eta_{p}^{2}=0.247$] on accuracy (%). *Post-hoc* comparisons revealed that participant accuracy was significantly higher when classifying the third repetition of a sound sequence (75.11 ± 10.22%) than when classifying the first (68.67 ± 12.15%) and second (70.89 ± 13.00%) repetitions.

**Figure 5:**
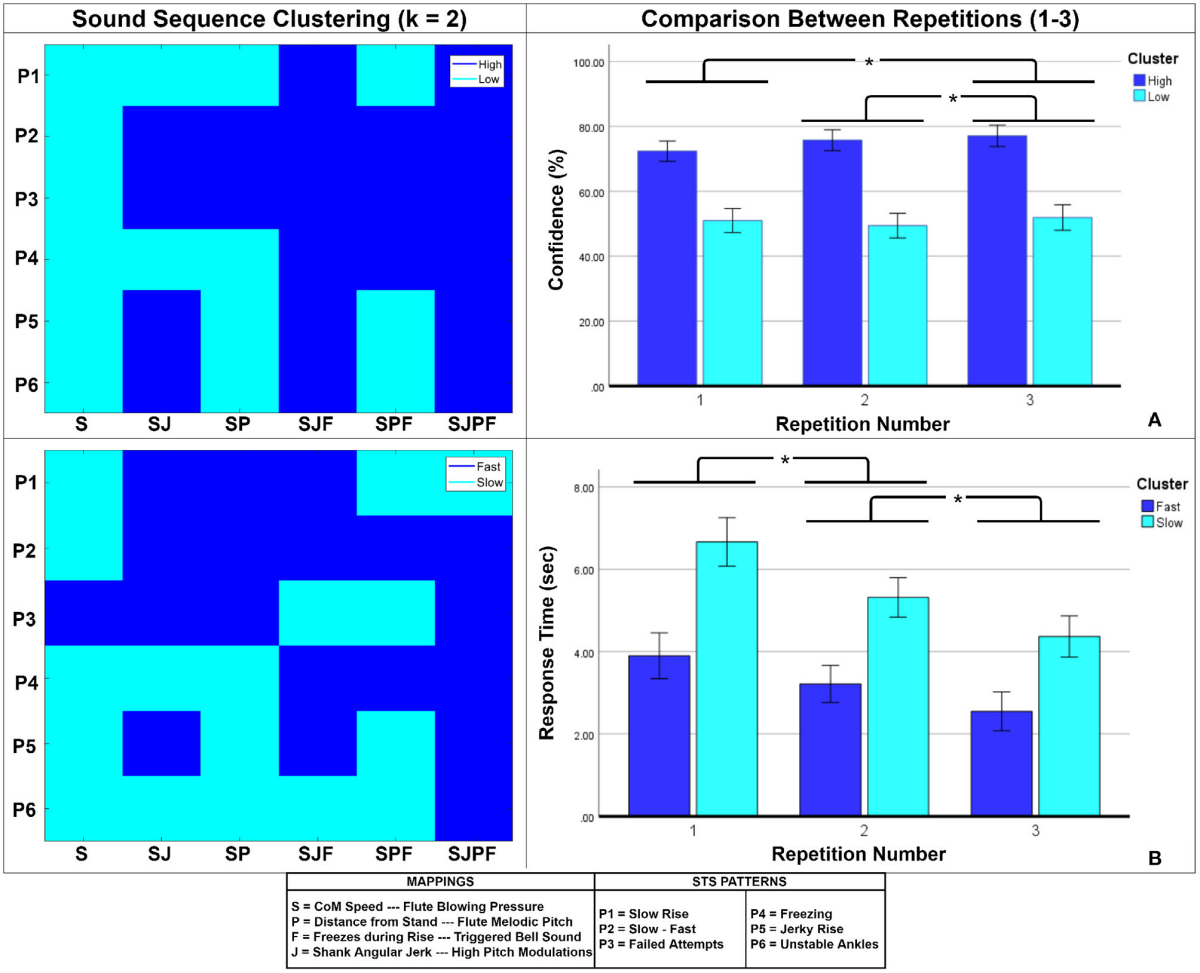
**(A)** Confidence-based clustering, **(B)** Response Time-based clustering of sound sequences. The clusters are shown on the left, and the comparison between repetition numbers is shown on the right. Bar heights indicate mean values, and the error bars show 95% confidence intervals. Asterisks indicate significant pairwise differences.

For confidence and response time, the results of the k-means clustering of sound sequences are shown on the left side of Figure 5. The *confidence* clusters appear well-defined, with the *high* cluster concentrated among the higher-dimensional parameter combinations and *slow-fast (P2) and failed attempts (P3)* sequences. A comparable tendency is seen for *response time* clusters, with many *high* confidence cluster members overlapping with *fast* response cluster members and vice versa. We then checked for differences between these clusters, and how they were evolved over multiple repetitions. For confidence, the mixed ANOVA found significant main effects of *repetition number* [*F*_(2, 68)_ = 5.847, *p* = 0.005, $\eta_{p}^{2}=0.147$] and *Cluster* [*F*_(1, 34)_ = 115.89, *p* < 0.001, $\eta_{p}^{2}=0.989$], as well as a significant interaction between them [*F*_(2, 68)_ = 4.703, *p* = 0.014, $\eta_{p}^{2}=0.122$]. As seen on the right side of Figure 5A, the *high confidence* cluster showed a mild increasing trend, whilst the *low confidence* cluster did not. *Post-hoc* comparisons revealed that *repetition number* 3 (66.61 ± 14.58%) elicited significantly higher confidence than *repetition number* 2 (64.79 ± 14.98%) and 1 (63.46 ± 12.76%) (right side of Figure 5A). Response time is depicted on the right side of Figure 5B. Here, there were significant main effects of *repetition number* [*F*_(2, 68)_ = 36.259, *p* < 0.001, $\eta_{p}^{2}=0.516$] and *Cluster* [*F*_(1, 34)_ = 76.787, *p* < 0.001, $\eta_{p}^{2}=0.693$], but no significant interaction between them. *Post-hoc* comparisons revealed significant differences among all repetitions (shown on right side of Figure 5B). Both clusters showed decreasing trends with increasing repetitions.

### 5.4. Effect of Music Training

There was no significant correlation between participants' overall accuracies and their GMSI scores (ρ = 0.34, *p* = 0.097). The RM ANCOVA did not reveal a significant main effect of *GMSI Score* on accuracy [*F*_(1, 23)_ = 3.002, *p* = 0.097, $\eta_{p}^{2}=0.115$], confidence [*F*_(1, 23)_ = 0.881, *p* = 0.377, $\eta_{p}^{2}=0.034$] or response time [*F*_(1, 23)_ = 2.895, *p* = 0.102, $\eta_{p}^{2}=0.112$].

### 5.5. Differences Based on Response Correctness

The one-way ANOVA revealed a significant main effect of *Correctness* on confidence [*F*_(1, 27.944)_ = 117.057, *p* < 0.001, $\eta_{p}^{2}$=0.807]. A *post-hoc* comparison revealed that participants were significantly more confident during correct classification responses (+7.364 ± 43.16% relative to their mean) than incorrect ones (−19.895 ± 39.27% relative to their mean). A significant effect of *Correctness* was also seen for response time [*F*_(1, 28.588)_ = 119.874, *p* < 0.001, $\eta_{p}^{2}$=0.807]. A *post-hoc* comparison revealed that participants exhibited significantly faster response times during correct responses (−19.13 ± 92.24% relative to their mean) than during incorrect ones (+48.13 ± 136.03% relative to their mean).

## 6. General Discussion

In this study, we designed and developed an adaptable sonification model for STS feedback and monitoring applications. We evaluated its veridicality (as defined by Barrass and Kramer, 1999) via a forced-choice classification task experiment where participants had to identify simulated STS patterns from their sonified sequences. We hypothesized that increasing the number of information mappings would increase classification accuracy, confidence, and reduce response time (H1), and this was validated by the strong effect of *Parameter Combination* on these outcomes. This finding aligns well with existing theories on categorization processes in the human brain, specifically that the uncertainty involved in assigning a category to a stimulus directly depends on the distance of the stimulus in perceptual space from “key representational features” of the category (Seger and Peterson, 2013). Thus, providing clear sonified information about these features appears to have resulted in less uncertainty, as is evidenced in the accuracy and confidence findings. The outcomes were also found to be highly pattern-dependent (validating H2), with two structurally distinctive patterns *(slow-fast, failed attempts)* being classified with greater accuracy and confidence than the others. Furthermore, there were distinct learning effects brought about by repeated exposure to sound sequences (effects of *Repetition Number*), but these did not transcend parameter combinations (no effect of *Block*), partly validating H3. This fits the theory that ambiguity in categorization processes decreases, and certainty increases as learning progresses (Seger and Peterson, 2013).

Even though four streams of information were presented simultaneously, our findings indicate that the participants were able to interpret the underlying information without major perceptual difficulties or cognitive overload (as evidenced by the *SPJF* accuracy figure of 86.67 ± 14.69%, which was well above the 16.67% chance level figure). Furthermore, this level of performance was achieved despite the relatively low fidelity of the sound reproduction system, showing that the model is robust to acoustical degradation. We credit this to the metaphorical and naturalistic sonification design (Antle et al., 2009; Roddy and Bridges, 2020), which leveraged existing embodied associations related to melody and dynamics in the representation of kinematic quantities. However, future studies should validate this by comparing the model with more abstract, non-metaphorical designs. It is also unclear at this point what level of detection accuracy is clinically usable, and how our obtained value should be interpreted in these terms. Clinical usability studies will have to be carried out in order to determine this.

### 6.1. The Sonification Model Components

The model comprised a *fixed component* to represent the rising motion itself (mappings S, P) and a *flexible component* to sonify kinematic variables of clinical interest—here we chose to represent freezes and shank jerk. The experiment results indicate that both components, as well as their individual constituent mappings, contributed positively to the classification process as seen in Figure 3A. This was interestingly at odds with the findings of Vinken et al. (2013), who found no effect of increasing the mapping dimensionality in a similar experiment. The difference in our case was most likely because the STS pattern characteristics and mapping rules were clearly conveyed to the participants. We now appraise both the model components.

**Fixed Component:** This represented the upward rising motion as a melody whose dynamic properties were controlled by the CoM speed. It seems reasonable to believe that its constituent variables are generalizable to any STS pattern type as their underlying assumption of the CoM moving with finite speed from sitting to standing coordinate positions (both modifiable) will always hold true. The fixed component by itself *(SP)* allowed a mean accuracy (%) of 57.11% (well above chance level). Its confusion matrix shows that true P2 (slow start-fast end) and P3 (failed attempts) sequences were classified with relatively few misclassifications. This can be attributed to the nature of the patterns and their resulting sound sequences; both pattern sequences had very distinctive amplitude envelopes and melodic contours (see Figure 1). It shows that participants were able to cognitively process the ordering of the melodic representation to interpret the movement trajectory at a coarse level, supporting the melodic sonification model for STS proposed by Newbold et al. (2015) and in line with the findings of Dyer et al. (2017). It is interesting to note that the addition of the melody mapping *(P)* did not result in significant improvements in classification accuracy over identical parameter combinations without it (e.g., *S* vs. *SP, SJF* vs. *SJPF*—see Figure 3A). However, it did lead to significantly higher confidence in both the above cases (see Figure 3C), implying that the presence of the melody helped strengthen the mental representation generated by the other feature mappings through its metaphorical depiction of spatial position. The use of melody for this purpose is supported by past findings of how spatial gestures are cognitively assigned to melodic contours (Kelkar and Jensenius, 2018).

The fixed component can also indirectly convey clinically relevant parameters through the melodic representation, such as transition duration (time taken to execute STS) and the speeds at which the different STS movement phases take place (Millor et al., 2014). The former is simply the time taken to traverse the entire musical scale, whilst the latter is apparent from the variation of scale note durations from start to finish. The same cannot be said for the more rapidly-varying parameters such as acceleration and jerk, and our experiment results indicate that the fixed component had clear limitations in this regard. The *SP* confusion matrix (see Figure 4, middle) shows a large number of misclassifications among P4, P5, and P6. This was expected, considering that *SP* did not contain the freezing and shank jerk information critical for identifying and disambiguating these patterns. Moreover, the *CoM Speed* feature was smoothed prior to mapping, and the melodic pitch mapping was essentially a spatial quantization of the rising motion. Although these steps were necessary to create a smooth-sounding and simple melodic sequence, they resulted in a loss of spatial and temporal resolution, making it harder to hear and understand fast changes in CoM speed that impaired STS patterns commonly exhibit (Scarborough et al., 2007; Nicolai et al., 2010; Peng et al., 2011; Millor et al., 2014; Boukadida et al., 2015). Even if CoM speed smoothing had been absent, the integration time of human loudness perception (Florentine et al., 1996) would have made this mapping inappropriate for representing rapid changes in speed.

The experiment also uncovered some flaws in the technical implementation of the melody mapping. The confusion matrix of *SJPF* (Figure 4, right) shows that a relatively large number of true P1 *(slow rise)* sequences were misclassified as P2 *(slow-fast)*. A closer examination of the *Distance from Stand* plot for P1 shows that its trajectory was non-linear. There seems to have been a relatively dominant contribution of the vertical component of STS displacement to the Euclidean distance variable, which caused the melody to accelerate after initial trunk flexion. This may have given the impression of a slow beginning and fast end to participants who did not receive the *slow-fast* sequence before the *slow rise* in the experiment order. The mapping function shape should be adjusted to ensure that normal rises result in melodies with temporally equi-spaced notes, so as to maintain the integrity of the conceptual metaphor. Another issue was that the final note of the ascending melody appears to have been triggered well before the rise was complete [see second row of Figure 1, where the feature value is 0.2 when the final note is triggered (last vertical dotted line)]. This highlights the importance of an accurate calibration procedure and suitable mapping shape function in clinical use-cases.

**Flexible Component:** In our experiment, the flexible component (*Freezing, Jerk* mappings) was able to effectively fill in the information gaps present in the fixed component, greatly improving the classification accuracy of the *Freezing, Jerky Rise, and Unstable Ankles* patterns as is clearly evident in Figure 4 (left) and Figure 3A. This shows that participants could clearly perceive and understand the flexible component mappings in parallel with the fixed component, which was expected given the clear spectral separation between the flute and the bell/high pitch modulations (Figure 2). Our findings support the notion that these sonifications were able to preserve the underlying structure of the information (Eldridge, 2006), allowing correct inferences to be drawn about the physical phenomenon (Bakogiannis et al., 2021).

We acknowledge that the flexible component was designed specifically based on our movement material and thus may not be universally generalizable, but propose, based on our results, that the same sonic entities (bell sound, high pitch modulations) can be used to, respectively, represent other movement features of clinical interest (e.g., mediolateral trunk tilt in stroke patients could be mapped to the high pitch modulations). Using the 3-segment STS computational model with a sonification framework such as that developed by Kantan et al. (2021) facilitates the realization of a wide range of such mappings for varied situations. Although some relevant movement features have been documented and measured using inertial sensors in the past (Millor et al., 2014), not all of them are amenable for real-time sonification, and future research must clearly define sonifiable features for different types of patients and impairments.

A key distinction between our movement materials and real-life pathological STS pattern categories is that the latter are likely to be less *clear-cut*, manifesting as combinations of discrete impairments resulting from muscle weakness and/or spasticity (Cheng et al., 1998; Scarborough et al., 2007; Boukadida et al., 2015). The sonification design strongly depends on the use-case scenario within the context of feedback and monitoring (Hildebrandt et al., 2014; Millor et al., 2014; Bresin et al., 2020). For example, a sonification of *jerk* may be useful for a therapist who is monitoring/assessing a patient, but not for feedback purposes, as patients may not be able to spontaneously convert this information into meaningful biomechanical change. It should be noted that although the results indicate that the freezing and jerk mappings were sufficiently salient, the participants were young and cognitively unimpaired. Future studies will investigate whether patients with varied degrees of cognitive impairment can perceive and comprehend the sounds—particularly in the case of augmented feedback applications (Sigrist et al., 2012; Morone et al., 2021). It is also possible that these salient entities (e.g., bell sounds) cause unacceptable levels of alarm fatigue (Cvach, 2012) and sonic disturbance in clinical monitoring contexts (Roodaki et al., 2017; Aldana Blanco et al., 2020).

### 6.2. Conceptual Advantages of Model

We believe that our model has several advantages at the conceptual level. All its constituent movement features are easily computed in real-time without any windowing or segmentation of the inertial data. The upward trajectory of the body CoM is directly converted to a congruent auditory representation, providing continuous task-relevant stream of information that can potentially aid associative sensorimotor learning (Sigrist et al., 2012; Dyer et al., 2015; Morone et al., 2021) or serve as sensory substitution for deafferented individuals. The mapping of CoM speed to the dynamics of the flute model ensures that there is sound only during periods of motion, which has been recommended as a means to reduce auditory fatigue and sensory overload when the body is at rest (Roodaki et al., 2017). In general, our results showed that the use of both analogic and symbolic mappings (Eldridge, 2006) in the model created a comprehensible balance of continuous and discrete kinematic information. Last but not least, the flexible component of the model can be tailored to suit clinical needs in various STS use-cases. Most of the clinically relevant variables listed by Millor et al. (2014) (vertical velocity, acceleration, jerk, joint angular velocity) can be computed from the 3-segment sagittal plane STS model we used (Musić et al., 2008) and mapped to suitable sonic entities within the model.

### 6.3. Learning Effects and Impact of Music Training

An interesting finding was that the difference between musicians' and non-musicians' performance parameters was not significant for any of the outcomes, even though musicians have been shown to be better than non-musicians at tracking the direction of frequency changes in auditory displays (Neuhoff et al., 2002). This result can be interpreted as evidence that music training is not a prerequisite in order to be able to effectively use the sonification model. The lack of significance can also be attributed to the relatively small sample size and the convenience sampling of participants (not necessarily at GMSI scale extremes; Müllensiefen et al., 2014), as well as the fact that the GMSI does not take general auditory comprehension and memory abilities into account, both important for classification in this context (Seger and Peterson, 2013). As far as learning effects were concerned, we did not find these to be present over the course of the experiment (i.e., across blocks) but they were certainly present within blocks (across sound sequence repetitions). The former is at odds with the findings of Vinken et al. (2013), who found significant across-block learning effects. In our case, the strong effects of *parameter combination* may have offset and/or masked any lingering effects across blocks. However, *within-block* effects were clearly seen for the accuracy, confidence, and response time outcomes. This indicates that the participants generally got better at using a single parameter combination with repeated exposure, pointing to the learnability of the model (Eldridge, 2006). However, the sound sequences we used across repetitions were identical, whereas repeated movement patterns in real life are unlikely to be so due to motor variability (Bernstein, 1966). It is therefore unclear at this point how the resulting variations in the auditory stimuli will affect their ability to be unambiguously categorized, in other words the level of *generalization* required in order to carry out the task (Seger and Peterson, 2013). This can be tested in future perceptual tests by additionally evaluating the ability of participants to classify one or more novel STS patterns *not* included in the training phases (like the *generalization tests* done by Dotov and Froese, 2018).

The sound sequences could be separated into two highly distinct clusters based on confidence and response time. Examining Figure 5, it is clear that the outcome for each sound sequence depended on whether it contained salient representations of pattern-distinctive information. E.g., for P4 (freezing) all parameter combinations having the F (freezes during rise) feature mapping were in the *high confidence* cluster, and vice versa. The results also indicate that positive learning effects were exclusive to the sequences that sonified pattern-distinctive information, showing that the participants got better at extracting and using this information in their judgments. Response time was inversely linked to confidence, as known from existing neuroscientific models of categorical uncertainty (Grinband et al., 2006). Participants exhibited highly distinct levels of confidence depending on response correctness as well, indicating that they themselves had a good awareness of when their responses were likely to be incorrect, i.e., it was rare that they confidently and quickly submitted an incorrect response. This is in line with existing theory, specifically that uncertainty during categorization is directly related to the perceptual distance between a stimulus and the existing mental representation of the category assigned to it (Seger and Peterson, 2013).

### 6.4. Limitations

We acknowledge that our methods and materials had several limitations. For the fixed component, we chose the flute for its continuous excitation signal and mellow tone, but did not evaluate its aesthetic values. Personal preferences and tastes related to musical instruments and scales are likely to exert an influence upon whether users choose to use such a communication model (Barrass and Kramer, 1999; Parseihian et al., 2015). Moreover, it is also likely that a simple ascending melody becomes monotonous and/or predictable after many repetitions, reducing the novelty and motivational value of the feedback (Maes et al., 2016). A solution can be the integration of rhythmic and harmony-based elements into the model. However, it is important to strike a balance between veridicality of information communication and stimulus novelty/unpredictability. Future studies can also include control conditions (e.g., sine wave sonification) to comparatively evaluate the aesthetic appeal and auditory fatigue, along with the other classification-related outcomes.

Our movement materials attempted to sample existing normal and impaired movement patterns based on STS literature (Kerr et al., 1991; Riley et al., 1997; Cheng et al., 1998; Scarborough et al., 2007). However, these were recordings of a single healthy individual, and did not cover the breadth of existing STS impairments seen in literature (Riley et al., 1997; Cheng et al., 1998; Scarborough et al., 2007; Millor et al., 2014). A more robust approach would have been to use inertial data recordings obtained from patients with a range of disabilities. An example is Danna et al. (2015), who recorded writing patterns of real dysgraphia patients for use in their perceptual test. Future studies will adapt the model based on recorded data obtained from patients suffering from STS impairments. the STS kinematic model we used (Musić et al., 2008) did not include mediolateral asymmetry, which is common among stroke patients (Cheng et al., 1998; Boukadida et al., 2015) and hip/knee arthroplasty patients (Wang et al., 2019, 2021). Lastly, the Madgwick filtering algorithm we used for orientation estimation (Madgwick et al., 2011) is prone to drifting over time depending on its configuration.

The model evaluation was done through a perceptual test, which in essence was an “open-loop” setting with no connection between the listener and the movement. The results from this assessment may not be generalizable to a motor feedback application, which is “closed-loop”. It will be necessary to perform human experiments to assess the model in a closed-loop feedback setting (such as the protocol employed by Gholinezhad et al., 2021). A potential pitfall in the experiment design was the fact that a forced-choice paradigm may have made elimination strategies possible, although more continuous measures of movement quality such as those used by Danna et al. (2015) are subject to individual variability. Aside from this, we did not test how *intuitive* the sonification model was, as we gave the participants explicit information of the mappings. Future studies should include an “implicit” condition where no information about the mappings is provided, similar to Danna et al. (2015) and Vinken et al. (2013).

### 6.5. Conclusions

We designed and developed an STS sonification model for rehabilitative and monitoring applications. The results of the listening test experiment showed that the model was capable of effectively conveying kinematic information about a set of normal and simulated impaired movement patterns. We believe that the model has several conceptual advantages that will allow it to be adapted to concrete applications for motor feedback and clinical monitoring, although our materials and experimental methods had some limitations. Future studies will upgrade the model based on movement data obtained from real patients, and evaluate it in these specific use-cases, exploring the versatility of the flexible component as a tool to represent various clinically relevant kinematic parameters. On the whole, there is a need for STS sonification design research, and our model provides a clear conceptual and technical starting point in the quest to leverage the potential of the sonic medium in clinical STS applications.

## Data Availability Statement

The original contributions presented in the study are included in the article/Supplementary Material, further inquiries can be directed to the corresponding author/s.

## Ethics Statement

The studies involving human participants were reviewed and approved by Aalborg University's Research Ethics Committee. The patients/participants provided their written informed consent to participate in this study.

## Author Contributions

PK was responsible for system design, development, conducting the expert interviews and the final evaluation. ES and SD supervised the project and assisted with analysis. All authors contributed to the article and approved of the final manuscript.

## Funding

This work was partially funded by NordForsk's Nordic University Hub, Nordic Sound and Music Computing Network NordicSMC, project number 86892.

## Conflict of Interest

The authors declare that the research was conducted in the absence of any commercial or financial relationships that could be construed as a potential conflict of interest.

## Publisher's Note

All claims expressed in this article are solely those of the authors and do not necessarily represent those of their affiliated organizations, or those of the publisher, the editors and the reviewers. Any product that may be evaluated in this article, or claim that may be made by its manufacturer, is not guaranteed or endorsed by the publisher.

## Footnotes

^1^JUCE Framework—https://juce.com/

^2^FAUST Programming Language—https://faust.grame.fr/

^3^ https://invensense.tdk.com/products/motion-tracking/9-axis/mpu-9250/

^4^Interview Material: https://docs.google.com/document/d/1VzfjOKnoHe1aWlH55eDd3Auxdzals8qJxaILIRadDVY/edit?usp=sharing

^5^Refer to the “Tutorial—STS Patterns” folder: https://www.dropbox.com/sh/e6di622gjmepo5s/AADxAN3hr6F3UpE4lafP1yTva?dl=0

^6^STS Experiment Audio Clips: https://www.dropbox.com/sh/e6di622gjmepo5s/AADxAN3hr6F3UpE4lafP1yTva?dl=0

^7^Goldsmith's Musical Sophistication Index—https://shiny.gold-msi.org/longgold_demo/?test=GMS&language=en

^8^Experiment Git Repository: https://github.com/prithviKantanAAU/FrontiersSTS_Code_Analysis
