# Supplementary material for: An Embodied Sonification Model for Sit-to-Stand Transfers
Source: Front Psychol. 2022 Feb 17;13:806861. doi: 10.3389/fpsyg.2022.806861 (PMC8891127; doi:10.3389/fpsyg.2022.806861)
Supplement: Supplementary file 1 [file Data_Sheet_1.pdf]

Supplementary Material

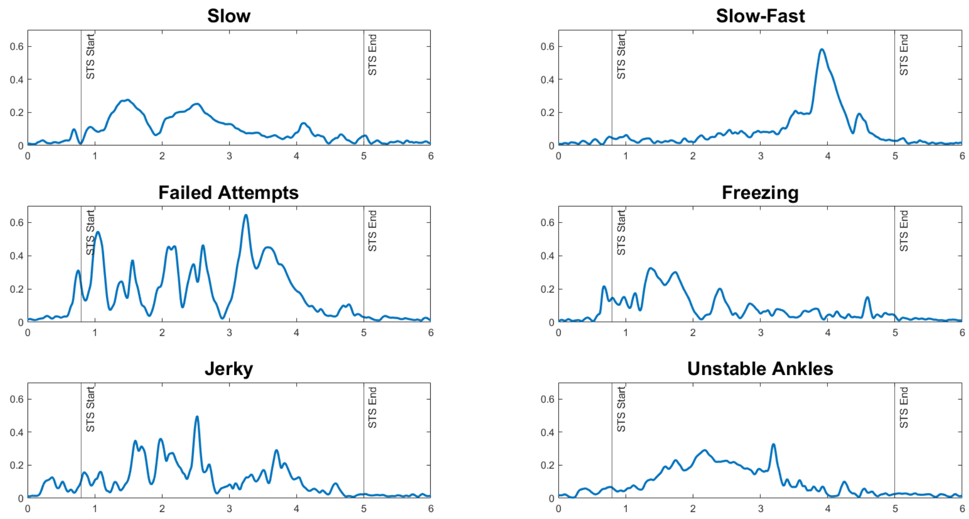

Figure S1. CoM Speed plots for the six STS patterns. The vertical axis represents CoM speed in bodylengths/sec, and the horizontal axis is time. The feature value before and after the rising motion is close to zero. Between these time bounds, the *slow-fast* and *failed attempts* patterns have distinctive contour shapes.

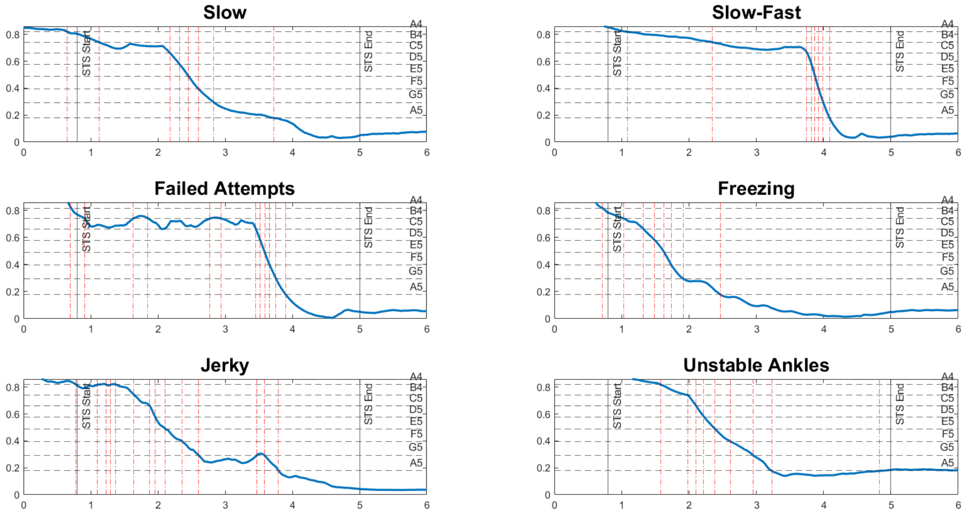

Figure S2. Distance from Stand plots for the six STS patterns. The vertical axis represents Distance from Stand, and the horizontal axis is time. The feature value decreases as the CoM reaches the 'stand' coordinates. The *slow-fast* and *failed attempts* patterns have distinctive contour shapes, the former has a gradual initial slope followed by sudden steepening, whilst the latter exhibits multiple oscillations prior to the rise. The *slow rise* pattern contour is *not* linear, even though it represents a normal STS transfer.

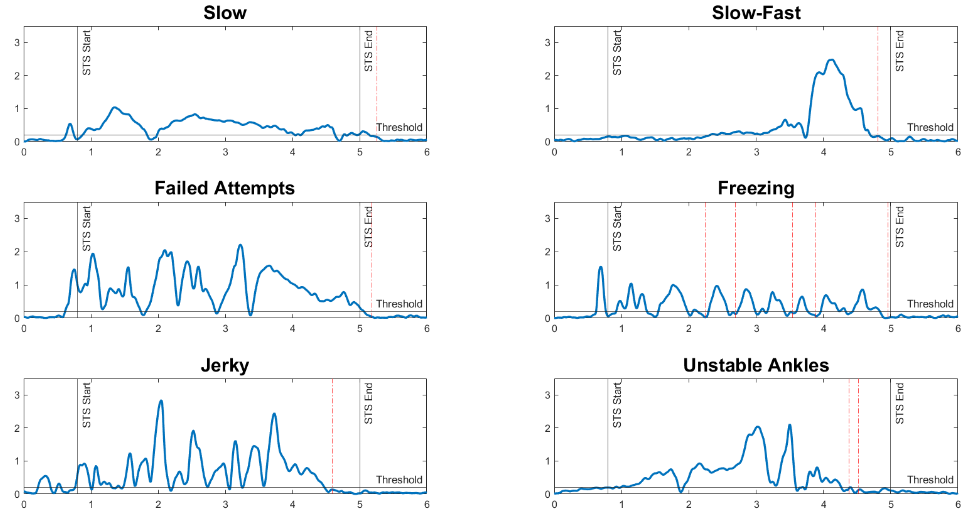

Figure S3. Hip angular velocity plots for the six STS patterns. The vertical axis represents angular velocity in $degrees/sec$, and the horizontal axis is time. The horizontal line 'Threshold' represents the velocity threshold under which the body is considered to be at rest. The dashed lines represent detected 'freezing' instants. All patterns register a freeze at the end of the STS transfer, but the *freezing* pattern is the only one that registers multiple freezes during the motion.

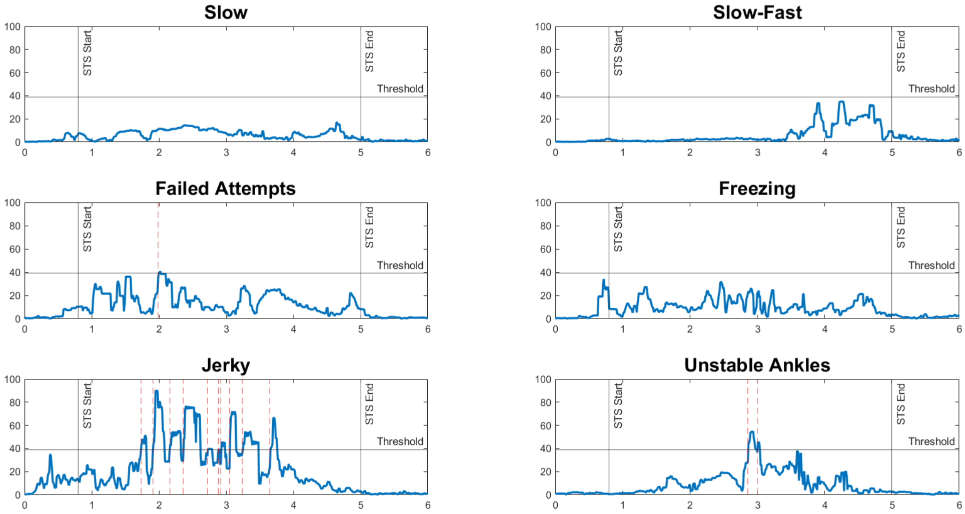

Figure S4. Shank angular jerk plots for the six STS patterns. The vertical axis represents shank angular jerk in $degrees/sec^3$, and the horizontal axis is time. The horizontal line ‘Threshold’ represents the maximum permissible jerk threshold. The dashed lines represent positive threshold crossings. The *failed attempts, jerky rise, and unstable ankles* patterns exhibit distinct contours in terms of the temporal locations and extents of the jerk threshold overshoots.

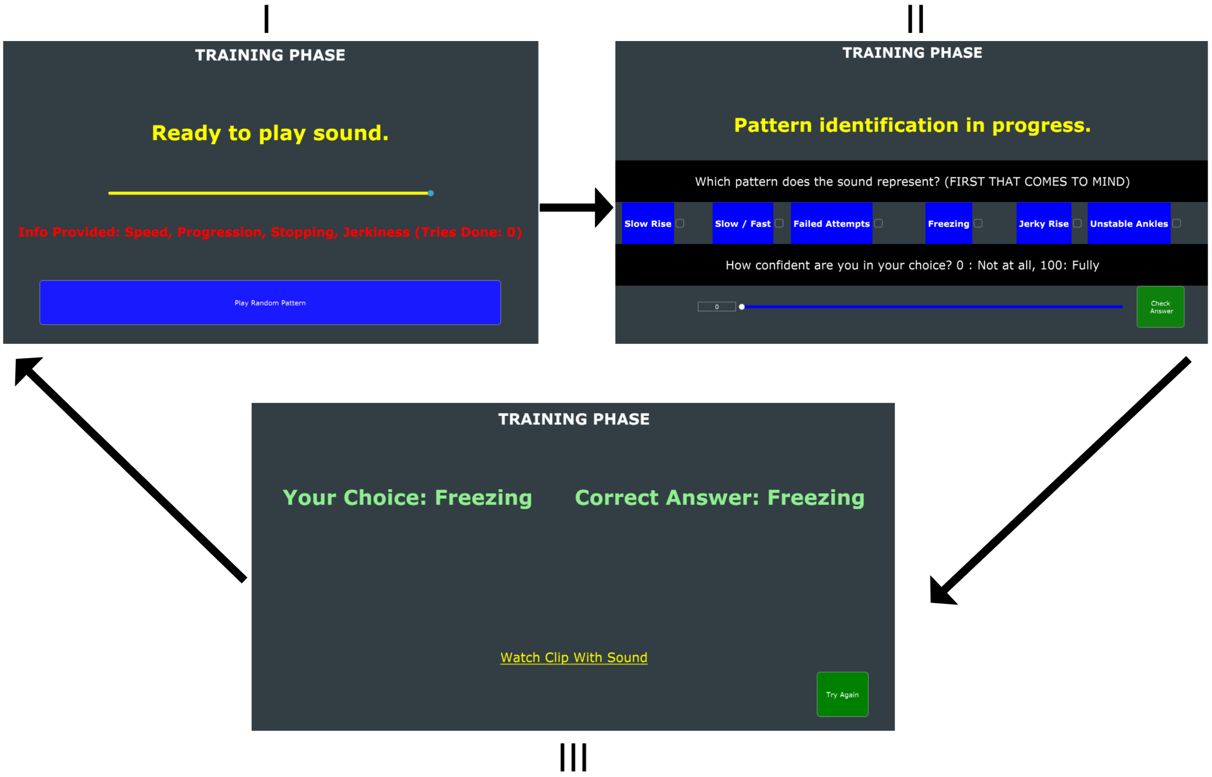

Figure S5. Depiction of the reinforcement learning phase. (I) The trial initiation screen. (II) The classification task screen, where participants were made to choose an STS pattern after listening to the sound sequence, and rate their confidence. (III) The feedback screen, where participants were told what the correct STS pattern was, and allowed to watch a the video clip in sync with the sound sequence.

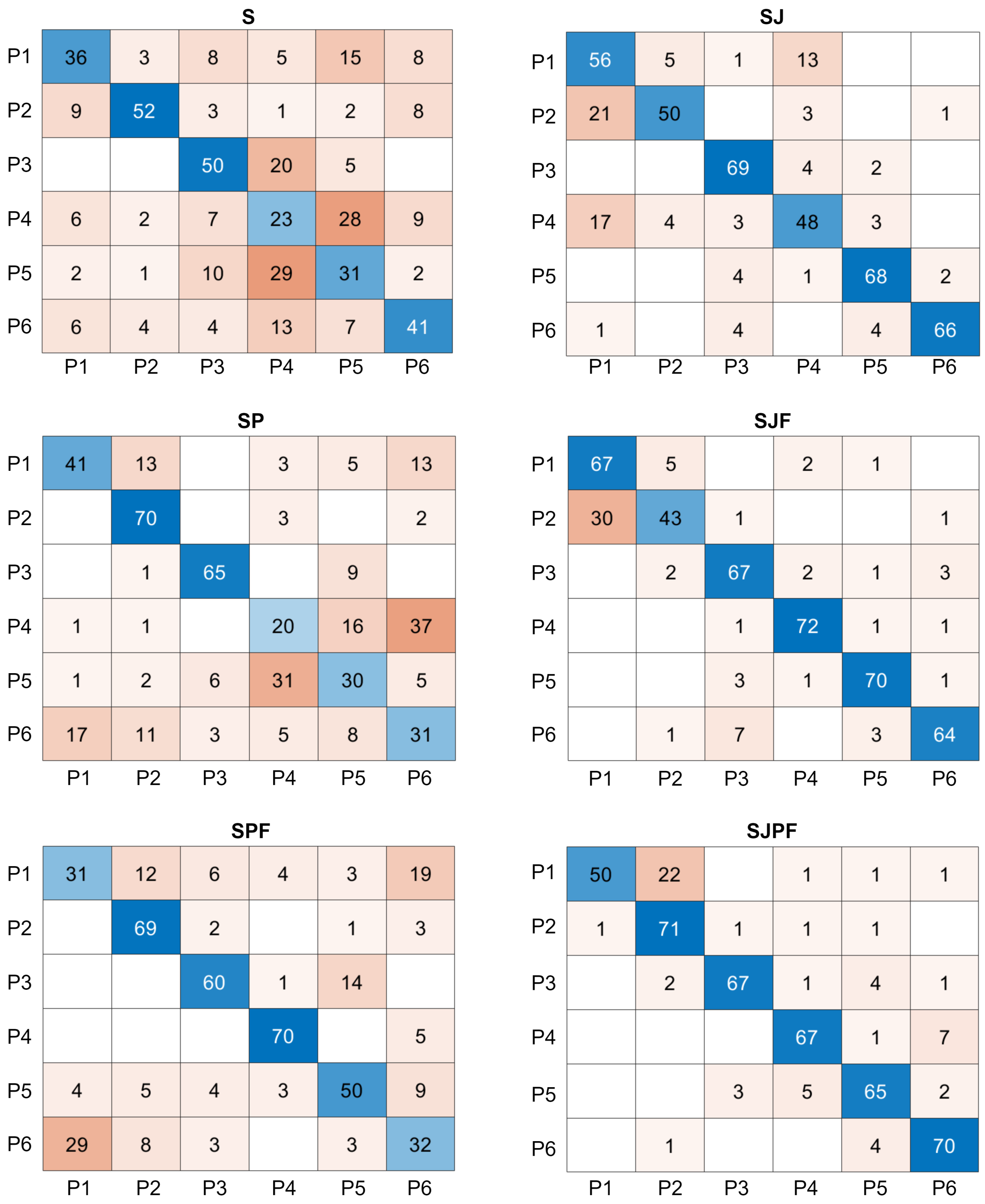

Figure S6. Confusion matrices for each of the six parameter combinations. Rows correspond to the true class, Diagonal elements represent correct classifications, and non-diagonal elements represent misclassifications.
